# Kinetic Spectrophotometric Method for The Determination of Ketoprofen in Pharmaceuticals and Biological Fluids

**Published:** 2006-12

**Authors:** A. El-Brashy, M. Eid, W. Talaat

**Affiliations:** *Department of Analytical Chemistry, Faculty of Pharmacy, Mansorua University, Mansoura 35516, Egypt*

**Keywords:** kinetic, spectrophotometry, ketoprofen, pharmaceutical preparations, biological fluids

## Abstract

A simple and sensitive kinetic method is described for the determination of ketoprofen in pure form, pharmaceuticals and biological fluids. The method utilizes an oxidative- coupling reaction based upon oxidation of 3-methyl-2-benzo-thiazolinone hydrazone hydrochloride (MBTH) with Ce(IV) in presence of HCl, where an electrophilic intermediate (diazonium salt of the reagent) is produced, then couples with ketoprofen yielding a highly colored condensation product. The absorbance is measured after 20 min at 605 nm. Calibration graph was linear over the concentration range of 1-8μg/mL with a minimum detection limit of 0.07 μg/mL. The proposed method was applied successfully to the determination of Ketoprofen in pharmaceutical preparations, plasma and urine. The % recoveries were 100.11 for pure form, 100.10 for tablets and gel, 100.0 for suspension and suppositories, 100.2 for capsules and ampoules and 99.79, 99.9 for plasma and urine. The results obtained were in good agreement with those obtained using reference methods for comparison.

## INTRODUCTION

**Figure 1:**
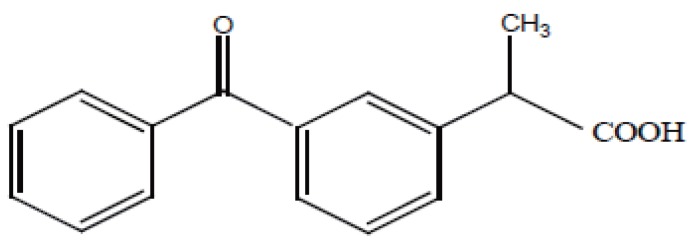
Structure of Ketoprofen.

RS-2-(3-benzoyl phenyl) propionic acid, Ketoprofen® (Fig. 1), is a non steroidal anti-inflammatory drug (1). It is used in musculoskeletal, joint disorders, dysmenorrheal, postoperative pain, gout and reduce fever (2). Ketoprofen has been analysed by different methods including HPLC (3-10), high performance frontal analysis (11), flow injection analysis (12), GC-MS (13-17), capillary zone electrophoresis (18), capillary electrochromatography (19, 20), colorimetry (21-24), spectrophotometry (25-27).

3-Methyl-2-benzothiazolinone hydrazone hydrochloride (MBTH) is one of the widly used chromogenic reagents for spectrophotometric analysis of phenols (28). It undergoes an interesting reaction with phenolic, amino, ketonic and aldehydic compounds in the presence of oxidizing agent such as H_2_O_2_, cerium (IV), iron (III), chromium (VI) yielding a highly colored reaction products (28). MBTH had been used for spectrophotometric determination of caffeine and theophylline (29), cefprozil (30), amoxicillin (31) and certian 4-quinolones in drug formulations (32).

Kinetic methods have certain advantages in pharmaceutical analysis regarding selectivity and elimination of additive interferences, which affect direct spectrophotometeric methods. The literature is still lacking in analytical procedures based on kinetic study for the determination of ketoprofen. In this work, the reaction between MBTH and ketoprofen was kinetically studied in an attempt to develop a reliable and specific spectrophotometric method for the determination of ketoprofen in pure form, pharmaceutical preparations, plasma and urine. The method is based on oxidation of MBTH with Ce (IV) then coupling with ketoprofen in presence of HCl, the colored condensation product is measured at 605 nm kinetically using the fixed time method.

## EXPERIMENTAL

### Apparatus

The spectrophotometric measurements were made with a Shimadzu (Kyoto, Japan) Model UV- 1601, UV- Visible recording spectrophotometer (P/N 206-67001), equipped with a kinetic accessory provided with temperature-controlled cells (TCC-240 thermoelectrically temperature). Recording range 0-1; wavelength 605 nm; factor 1; number of cells 1; reaction time, 20; cycle time 0.1 min.

### Materials and reagent solutions

Ketoprofon was obtained as a gift and used as received from Sigma Pharmaceuticals, Egypt. 3-Methyl benzothiazolin-2 one hydrazone (Sigma, st. lousis, MO). 0.3% w/v aqueous solution was freshly prepared every day.

Cerium (IV) ammonim sulphate 1.25% w/v was prepared in 0.25% w/v H_2_SO_4_. Hydrochloric acid, 2M aqueous solution. Sulphuric acid, 0.25% w/v aqueous solution and ethanol. All were of analytical reagent grade of Merck (Darmstadt, Germany).

Different dosage forms were commercially purchased from different markets.

### Preparation of standard stock solutions

An accurately weighed amount (25 mg) of ketoprofen was transferred into a- 50 ml volumetric flask and dissolved in 20 ml ethanol then completed to the mark to provide a stock solution containing 500 μg/mL. The solution was kept at 4°C until using, and diluted with ethanol to obtain the suitable working concentrations.

### General assay procedure

Transfer aliquots of the standard stock solution of ketoprofen within the concentration range 1-8 μg/mL into a-10 ml calibrated flask. Add 1.5 mL of 0.3% w/v MBTH aqueous solution, 1 mL of 2 M HCl, 1 mL of 1.25% w/v Ce (IV) ammonium sulphate and dilute to volume with distilled water. Measure the absorbance after 20 min at 605 nm vs. reagent blank prepared simultaneously. Construct the calibration graph by plotting the final concentration of the drug in μg/mL vs. the absorbance values. Alternatively, derive the corresponding regression equation at the specified fixed time.

### Procedure for pharmaceutical preparations

**Tablets and Capsules:** Weigh, accurately an amount of pulverized 10 tablets or mixed 10 capsules equivalent to 25 mg ketoprofen, extract with ethanol, filter if necessary and complete to 50 mL with the same solvent. Transfer aliquots of these solutions into 10 mL volumetric flasks and proceed as under the general procedure. Calculate the nominal concentration from the calibration graph previously prepared.

**Ampoules:** Transfer an aliquot of the mixed three ampoules equivalent to 25 mg of Ketoprofen. Extract with 25 mL ethyl acetate, add 1 mL of 2 M HCl, and evaporate the organic layer under a stream of nitrogen. Dissolve the residue in ethanol, dilute to 50 mL with the same solvent in a volumetric flask, and proceed as under the general procedure. Calculate the percentage recovery using a calibration graph previously prepared.

**Suspension:** Transfer an aliquot of the suspension equivalent to 25 mg of Ketoprofen. Extract with 25 mL ethyl acetate, evaporate the organic layer under a stream of nitrogen. Dissolve the residue in ethanol, dilute to 50 mL with the same solvent in a volumetric flask, and proceed as under the general procedure. Calculate the percentage recovery using a calibration graph previously prepared.

**Suppository:** Weigh five suppositories, transfer to a porcelain dish, melt and allow to cool while stirring with a glass rod. Accurately weigh a portion of the melted suppository mass, equivalent to 100 mg of ketoprofen, extract with 25 mL ethanol, filter, dilute the filtrate to 100 mL with the same solvent, and proceed as described under the general assay procedure.

**Gel:** Accurately weigh 1 gm of the gel equivalent to 25 mg of ketoprofen. Extract with 25 mL of ethyl acetate, sonicate for 20 min, filter, evaporate the organic layer under a stream of nitrogen gas. Dissolve the residue in ethanol, dilute to 50 mL with the same solvent and proceed as descried under the general procedure.

### Procedure for biological fluids

Aliquots of serum or urine (1.0 mL), were spiked with ketoprofen, acidified with HCl, then extracted with 5 mL ether, vortxed and centrifuged. The organic layer was evaporated under a stream of nitrogen gas. The residue was dissolved in ethanol. This solution was transferred quantitatively into a series of 10 mL volumetric flasks and proceeds as described under the general procedure. A blank experiment simultaneously was performed.

## RESULTS AND DISCUSSION

**Figure 2:**
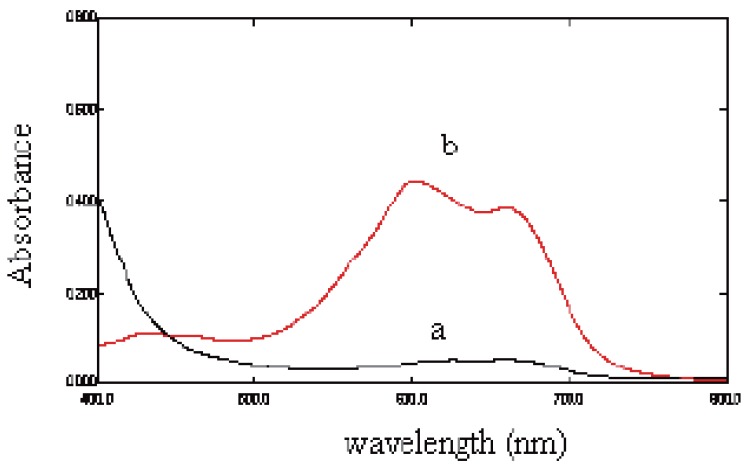
Absorption spectra of (a) MBTH (0.3%) and (b) The reaction product of ketoprofen (4 μg/mL).

MBTH is oxidized with Ce (IV) then coupling with ketoprofen in presence of HCl, the colored condensation product measured at 605 nm kinetically using the fixed time method (Fig. 2).

### Optimization of reaction conditions

**Effect of concentration of MBTH.** The effect of concentration of MBTH solution was investigated by carrying out the reaction using different concentrations of MBTH ranging from 0.2%-0.5% (w/v). The maximum absorbance was obtained upon using 1.5 ml of 0.3% (w/v) MBTH solution.

**Effect of concentration of Hydrochloric acid.** The molarity of HCl was investigated by carrying out the reaction using different molarities ranging from 1 - 2.75 M. 1.0 mL of 2M HCl was satisfactory for the reaction.

**Effect of concentration of Ce (IV) ammonium sulphate solution.** The effect of concentration of Ce (IV) ammonium sulphate solution was studied by carrying out the reaction using different concentrations of Ce (IV) ammonium sulphate solution ranging from 1%-2% (w/v). An increase of absorbance was obtained upon using 1.0ml of 1.25% (w/v) Ce (IV) ammonium sulphate solution.

**Effect of concentration of H_2_SO_4_.** The optimum concentration of H_2_SO_4_ was 0.25% (w/v) as solvent for Ce(IV).

### Kinetics of the reaction

**Figure 3:**
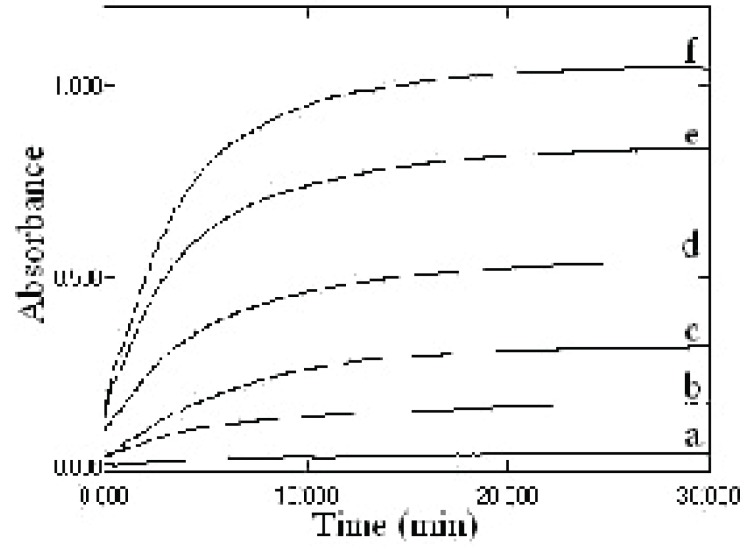
Effect of time on the absorption of different concentrations of ketoprofen. (a), blank; (b), 1 μg/mL; (c), 2 μg/mL; (d), 4 μg/mL; (e), 6 μg/mL; (e), 8 μg/mL.

**Figure 4:**
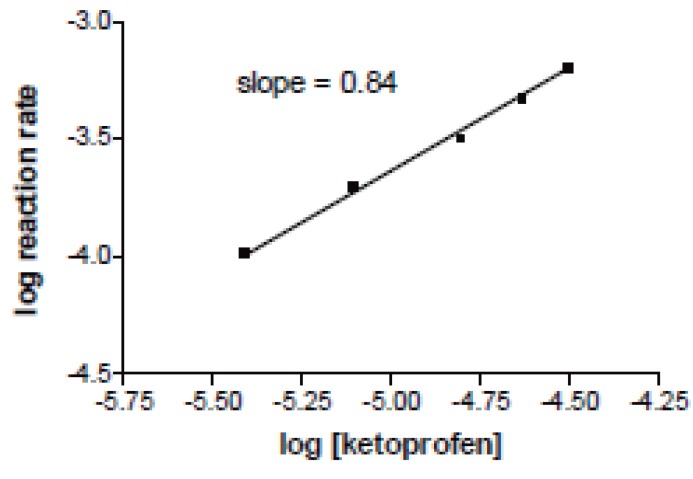
A plot of log reaction rate versus log concentration of ketoprofen.

Because the intensity of the color increased with time (Fig. 3), this was used as the basis for a useful kinetic method for the determination of ketoprofen, the rate of the reaction was monitored with various concentrations of ketoprofen over the range of 1-8 μg/mL, the graph shown in Figure 4 indicates that the reaction rate of ketoprofen obeys the following equation:

Rate = K’ [Drug]^n^

Where K’ is the rate constant and n is the order of the reaction. The rate of the reaction may be calculated by variable time method measurement (33, 34) as ΔA/Δt, A is the absorbance and t is the time in seconds (Table 1). By taking logarithms of rates and concentration (Fig 3), equation 1 is transformed into:

Log rate = log ΔA/Δt = Log K’ + n Log [drug]

Log rate = –3.94 + 0.82 log [ketoprofen] (r=0.9920)

where r is the correlation coefficient.

**Table 1:** Logarithms of rates for different concentrations ketoprofen at room temperature

Log rate ΔA/Δt	Log [ketoprofen]

-3.98	-5.41
-3.70	-5.10
-3.58	-4.80
-3.35	-4.63
-3.20	-4.50

Thus K’ = 6.31 × 10^-3^S^-1^, and the reaction is first order (n=0.82) with respect to ketoprofen concentration.

**Evaluation of kinetic methods.** Several experiments were run to obtain ketoprofen concentration using the rate data, rate constant, fixed-concentration and fixed time methods (33, 34), and the most suitable analytical method was selected taking into account the applicability and sensitivity (the slope of the calibration graph, the correlation coefficient (r), and the intercept).

**Figure 5:**
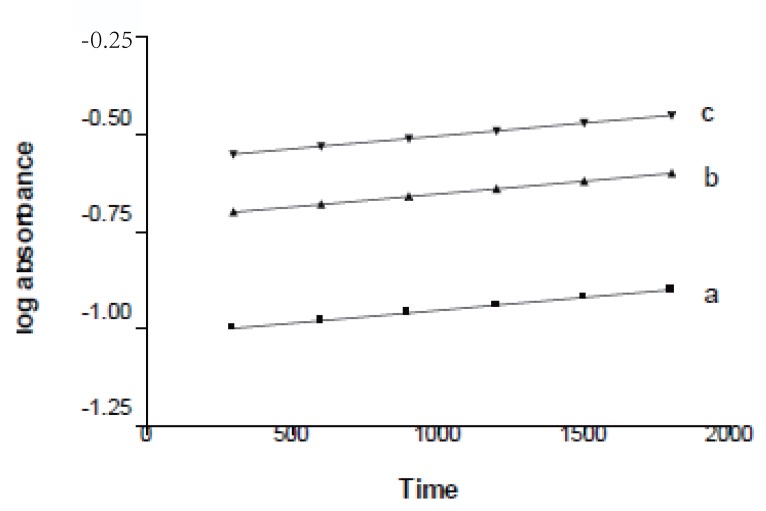
A plot of logarithm absorbance versus time for different concentrations of ketoprofen. a), 1 mg/mL; b), 2 mg/mL; c), 6 mg/mL.

**Rate constant method.** The best way to obtain an average K’ value for the reaction is to plot the logarithm of the concentration or the logarithm of any related property versus time. The slope of the line is -K/2.303, from which the rate constant is obtained (Table 2). Graph of log (A) versus time for ketoprofen in the concentration range 1-8 μg/mL was plotted and it appeared to be rectilinear (Fig. 5). Pseudo first order rate constant (K’) corresponding to different ketoprofen concentrations (C) were calculated from the slopes multiplied by -2.303.

**Table 2:** Values of K’ calculated from slopes of log A vs. t graphs at 605 nm

K’ (S^-1^)	[ketoprofen], M

-1.52 × 10^-4^	3.90 × 10^-4^
-1.84 × 10^-4^	7.87 × 10^-6^
-2.80 × 10^-4^	1.57 × 10^-5^
-1.83 × 10^-5^	2.36 × 10^-5^
-1.77 × 10^-4^	3.15 × 10^-5^

Regression of (C) versus K’ gave the equation:

K’ = –0.16 + 0.08C (r=0.5)

**Fixed absorbance method.** Reaction rate data were recorded for different ketoprofen concentrations in the range 1-8 μg/mL. A preselected value of the absorbance (0.49) was fixed and the time was measured in seconds (Table 3). The reciprocal of time (1/t) versus the initial concentration of ketoprofen was plotted and following equation of calibration graph was obtained:

1/t = –3.89×10^+3^+ 1.18×10^-3^C (r=0.9994)

**Table 3:** Values of reciprocal of time (1/t) taken at fixed absorbance (0.49) for different concentrations of ketoprofen at constant concentration of MBTH

1/t(s^-1^)	[ketoprofen], M

8.30 × 10^-4^	1.57 × 10^-5^
3.30 × 10^-3^	3.36 × 10^-5^
5.56 × 10^-3^	3.15 × 10^-5^

The concentration of MBTH=0.3%(w/v).

The range of ketoprofen concentrations giving the most satisfactory results was limited therefore this method was abandoned.

**Fixed time method.** Reaction rates were measured for different concentrations of ketoprofen ranging from 1-8 μg/mL. At a preselected fixed time, which was accurately determined, the absorbance was measured. Calibration graphs of absorbance versus initial concentration were established at fixed time of 5, 10, 15, 20, 25, and 30 min with the regression equation assembled in Table 4. It is clear that the slope increases with time and the most acceptable values of correlation coefficient (r) and the intercept were obtained at a fixed time of 20 min which was therefore chosen as the most suitable time interval for measurement. The percentage recoveries for the determination of ketoprofen in pure form by fixed time method are shown in Table 5.

**Table 4:** Regression equations for ketoprofen at fixed time at room temperature

Time (min)	Regression equation	Correlation coefficient (r)

5	A=0.025+0.088C	0.9900
10	A=0.005+0.109C	0.9980
15	A=0.131+0.0985C	0.9900
20	A=0.003+0.123C	0.9999
25	A=0.134+0.0994C	0.9990
30	A=0.18+0.0944C	0.9990

A, absorbance; C, concentration; r, correlation coefficient.

The results obtained with the proposed method and shown in Table 5 were compared to the official titrimetric method for ketoprofen (1) by means of *t*- and *F*-values at 95% confidence level (35). The average results obtained by proposed method and official method were statistically identical, as the difference between the average values had no significance at 95% confidence level.

**Table 5:** Determination of ketoprofen in its pure form using the proposed method

Proposed Method			Official Method (1)	
Amount taken (μg/mL)	Amount found (μg/mL)	% recovery	Amount taken (mg/mL)	% recovery^a^

1	1.00	100.00	200.00	100.00
2	2.01	100.50	300.00	100.20
4	4.01	100.25	400.00	100.10
6	6.00	100.00		
8	7.99	99.88		
Mean ± S.D		100.13 ± 0.25		100.11 ± 0.10
*t*- test		0.13	(1.94)^b^	
*F*- test		6.25	(19.16)^b^	

a Each result is the average of three separate experiments;

b The value of tabulated *t* and *F*, (at *p*=0.05) (36).

### Stoichiometry of the reaction

**Figure 6:**
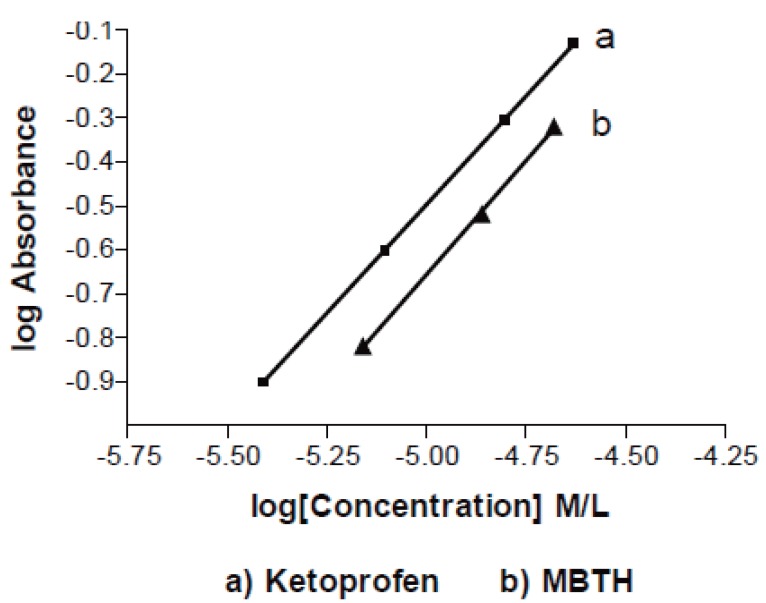
Determination of molar ratio by limiting logaritmic method. a), Ketoprofen; b), MBTH.

**Figure 7:**
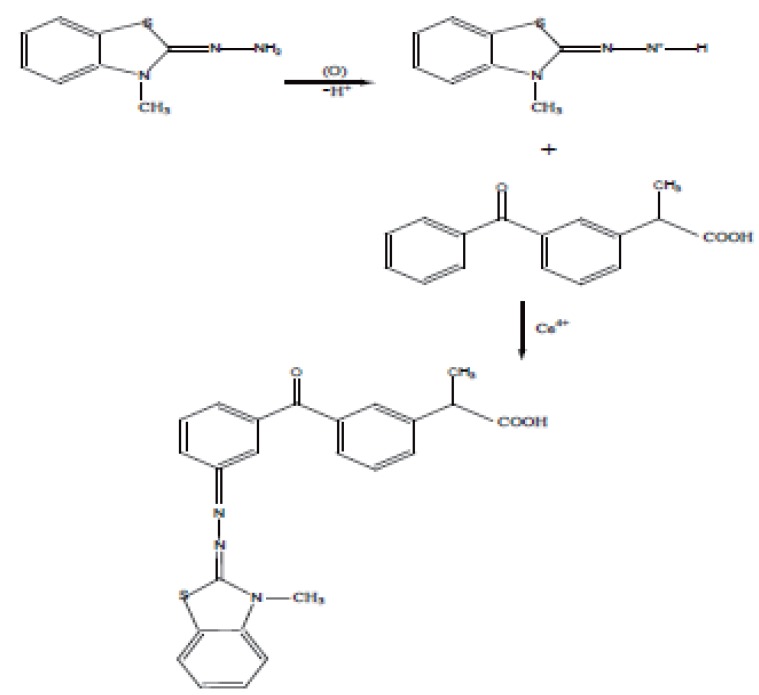
Proposal of the oxidative coupling reaction between MBTH and Ketoprofen.

The stoichiometry of the reaction was determined adopting limiting logarithmic method. A plot of log absorbance vs. log [MBTH] and log [ketoprofen] gave straight lines, the value of the slopes are 0.99 and 0.999 respectively (Fig. 6). The reaction proceeds in the ratio 1:1 as in Figure 7.

### Validation of the proposed method

**Linearity of the method.** After optimizing the conditions, it was found that the relation between the absorbance and final concentration was linear over the range of 1-8 μg/mL, linear regression analysis of the results gave the following equation:

A = 0.003 + 0.123C (r=0.9999)

where A=absorbance; C=Concentration in μg/mL; r=Correlation coefficient.

**Accuracy and Precision.** The precision of the proposed method was determined by replicate analysis of three concentrations of the standard solutions. The % relative standard deviation was 0.36. Statistical analysis (35) of the standard deviations of the residuals (S_y/x_) 8.6 × 10^-4^ and of slope (S_b_) 8.4 × 10^-4^, and of intercept (S_a_) 2.9 × 10^-3^; these small values indicate high precision of the method.

The results of intraday and interday accuracy and precision which indicate the repeatability and reproducibility of the proposed method are summarized in Table 6. The accuracy and precision of the proposed method are high as illustrated by the low values of SD, %RSD, low values of %Er.

**Table 6:** Evaluation of accuracy and precision data of the proposed kinetic spectrophotometric method for the determination of ketoprofen

Concentration Added (μg/mL)	Mean ± SD	% Recovery	% RSD	% Error

Intra-day				
2.0	2.002 ± 0.30	100.10	0.30	0.17
4.0	4.000 ± 0.20	100.00	0.20	0.12
Inter-day				
2.0	2.004 ± 0.40	100.20	0.40	0.23
4.0	4.012 ± 0.30	100.30	0.30	0.17

**Limit of detection and quantification.** The limit of detection (LOD) was calculated from the calibration curve according to LOD=3.3 S_a/b_ with S_a_ being the standard deviation of intercept of regression line and b being the slope of the calibration curve (35) and it was found to be 0.07 μg/mL.

The limit of quantification, defined here as LOQ=10 S_a/b_ was determined on the basis of standard deviation of response and the slope and it was found to be 0.23 μg/mL.

### Application to commercial formulations

The proposed method was successfully applied for the determination of ketoprofen in different dosage forms. The results shown in Table 7 are in good agreement with those obtained with the compendial methods, UV spectrophotometry (21) using hydroxyl amine hydrochloride as a reagent, colorimetric (22, 23) using 2-nitro phenyl hydrazine and methylene violet respectively, second derivative spectrophotometry (25).

**Table 7:** Determination of ketoproten in pharmaceutical preparations

Preparation	Proposed method			Compendial methods	
	Amount taken (μg/mL)	Amount found (μg/mL)	% recovery^a^	Amount taken (μg/mL)	% recovery^a^

1) ketofan capsules 50 mg ketoprofen/cap	1.0	0.998	99.80	100.0	100.00 (22)
	2.0	2.01	100.50	200.0	100.20
	4.0	4.01	100.25	300.0	100.30
	6.0	6.00	100.00		
Mean ± S.D			100.14 ± 0.30		100.20 ± 0.20
*t*- test			0.29	(2.05)^b^	
*F*- test			2.25	(19.16)^b^	
2) Ketofan tablets 25 mg ketoprofen/tab	1.0	1.00	100.00	5.00	100.00 (23)
	2.0	2.01	100.50	10.00	99.90
	4.0	4.02	100.50	15.00	100.40
	6.0	5.99	99.83		
Mean ± S.D			100.21 ± 0.34		100.10 ± 0.30
*t*- test			0.46	(2.05)^b^	
*F*- test			1.28	(19.16)^b^	
3) Dolokit ampoules 10 mg ketoprofen/amp	1.0	1.01	101.00	10.0	100.00 (21)
	2.0	2.00	100.00	20.0	99.80
	4.0	4.03	100.75	30.0	100.80
	6.0	5.99	99.83		
Mean ± S.D			100.39 ± 0.57		100.20 ± 0.53
*t*- test			0.47	(2.05)^b^	
*F*- test			1.15	(9.55)^b^	
4) Fastum gel 2.5% ketoprofen	1.0	1.01	101.00	0.5 mg/mL	100.00 (25)
	2.0	2.01	100.50	0.6 mg/mL	100.30
	4.0	3.99	99.75	0.7 mg/mL	99.90
	6.0	6.00	100.00		
Mean ± S.D			100.31 ± 0.55		100.10 ± 0.20
*t*- test			0.60	(2.05)^b^	
*F*- test			7.56	(19.16)^b^	
5) Ketofan Suspension 12.5 mg ketoprofen/mL	1.0	0.997	99.70	5.00	100.00 (23)
	2.0	2.01	100.50	10.00	99.80
	4.0	4.01	100.25	15.00	100.30
	6.0	6.00	100.00		
Means ± SD			100.11 ± 0.34		100.00 ± 0.30
*t*- test			0.46	(2.05)^b^	
*F*- test			1.28	(19.16)^b^	
6) Alcofan suppositories 100 mg ketoprofen/suppository	1.0	0.996	99.60	0.1	100.00 (22)
	2.0	2.01	100.50	0.2	99.90
	4.0	4.00	100.00	0.3	100.20
	6.0	5.99	99.83		
Mean ± S.D			99.98 ± 0.38		100.00 ± 0.20
*t*- test			0.10	(2.05)^b^	
*F*- test			3.61	(19.16)^b^	

a Each result is the average of three separate experiments;

b The value of tabulated *t* and *F*, (at *p*=0.05) (36).

1), Ameryia-Alexandria-Egypt (Batch No.440544); 2), Ameryia-Alexandria-Egypt (Batch No.918743); 3), Sigma Pharmaceuticals-Egypt (Batch No.963340); 4), Minapharm-Egypt (Batch No.1750); 5), Amryia-Alexandria-Egypt (Batch No.958534); 6), Alexandria pharmaceuticals-Alexandria-Egypt (Batch No.4901020).

### Application to biological fluids

Ketoprofen is readily absorbed from the gastro-intestinal tract; peak plasma concentrations occur about 0.5 to 2 hours after a dose. It is 99% bound to plasma proteins (2), so its concentration in plasma is in the working concentration range of the proposed method. The high sensitivity attained by the proposed method allows the determination of ketoprofen in biological fluids; Table 8 shows the results of recovery studies.

**Table 8:** Determination of ketoprofen in plasma and urine by the proposed method

Proposed Method			Official Method (1)	
Amount taken (μg/mL)	Amount found (μg/mL)	% recovery	Amount taken (mg/mL)	% recovery^a^

1	1.00	100.00	200.00	100.00
2	2.01	100.50	300.00	100.20
4	4.01	100.25	400.00	100.10
6	6.00	100.00		
8	7.99	99.88		
Mean ± S.D		100.13 ± 0.25		100.11 ± 0.10
t- test		0.13	(1.94)^b^	
F- test		6.25	(19.16)^b^	

a Each result is the average of three separate experiments;

b The value of tabulated t and F, (at *p*=0.05) (36)

## CONCLUSION

The proposed kinetic method is the first kinetic method for determination of ketoprofen. It is accurate, selective, and reproducible, requires simple apparatus for its performance. Moreover, small amounts are assayed with good results compared with the official method which needs not less than 100 mg. The proposed method is applied to determination of ketoprofen in different dosage forms. No significant difference obtained by the results of the proposed method and the compendial ones. Consequently, it is suitable for routine quality control of ketoprofen.
